# Supplementary material for: Are carbon emissions trading and green financial instruments synergistic? -Comprehensive quantitative research based on content analysis
Source: PLoS One. 2024 Mar 7;19(3):e0298601. doi: 10.1371/journal.pone.0298601 (PMC10919601; doi:10.1371/journal.pone.0298601)
Supplement: S1 Data — (ZIP) [file pone.0298601.s001.zip › Data/地级市Z_F工作报告.zip!/┴╔─■/╙¬┐┌╩╨/└┤╫╘╣1⁄2╓┌║┼í╛┬φ┐╦╩2╛▌═°í┐_2016.docx]

各位代表：

现在，我代表市政府向大会报告工作，请予审议，并请市政协委员和列席会议的同志提出意见。

一、“十二五”时期及2015年主要工作回顾

“十二五”时期，面对复杂多变的国内外形势，在省委、省政府和市委的正确领导下，在市人大、市政协的监督支持下，市政府组织带领全市人民，积极抢抓国家重大发展机遇，攻坚克难，拼搏进取，保持了经济持续平稳健康发展、社会和谐稳定的良好局面。

经济发展质量和效益同步提升。预计地区生产总值年均增长9%，人均地区生产总值年均增长8.7%，固定资产投资年均增长6.7%，社会消费品零售总额年均增长13.5%，城镇、农村常住居民人均可支配收入持续高于经济增速，年均分别增长11.6%、13%，一般公共预算收入中非税收入、涉土税收占比分别为16.8%和21.6%，比2010年分别下降0.1个和18个百分点。

产业结构更趋合理。粮食等农产品产量基本稳定，农业基础地位进一步巩固。冶金、装备制造等六大主导产业对工业经济的支撑作用日益凸显。战略性新兴产业和现代服务业快速发展，全市经济实现了由主要依靠工业向二、三产业共同拉动的转变。三次产业比例由2010年的7.7:55.3:37调整到2015年的7.3:48.1:44.6。

发展活力不断增强。全面深化各项改革，市本级行政职权减少3862项，减幅达到72%。对外开放水平进一步提升，利用外资结构不断优化，对外贸易快速发展，外贸出口累计实现206亿美元，是“十一五”时期的2.7倍，年均增长15.8%。港口货物吞吐量、集装箱运量年均分别增长8.4%、12.2%。

城乡面貌明显改观。稳步推进新型城镇化建设，常住人口城镇化率提高5.9个百分点，“一带三轴”多点支撑的城市发展格局初步形成。兰旗机场等一批重大基础设施项目建成，城市综合承载力进一步增强。生态文明建设取得成效，森林覆盖率提高1.6个百分点，城市污水处理率提高10个百分点，环境治理能力不断提升。我市荣获国家森林城市称号。

人民生活质量持续改善。累计投入民生和社会事业资金752亿元，一大批群众关心关注的热点、难点问题得到有效解决。覆盖城乡的社会保障体系逐步健全，保障性住房建设有序推进，城镇登记失业率均控制在3%以内。社会各项事业全面发展，营口理工学院设立，营口高等教育实现历史性突破。新农合管理经验向全省全国推广，文化和群众体育工作荣获全国文化、体育系统先进称号。

在刚刚过去的2015年，我们坚持稳中求进总基调，坚持一张蓝图绘到底，保持工作的连续性和稳定性，统筹稳增长、促改革、调结构、惠民生、防风险，精准发力，主动作为，不仅实现了“十二五”规划的良好收官，也为“十三五”时期发展打下了坚实基础。

按照省委、省政府的要求，本着实事求是、求真务实的原则，在夯实各项经济数据之后，2015年地区生产总值实现1513.8亿元，增长4.5%；固定资产投资906.2亿元，下降22%；一般公共预算收入104.2亿元，下降35%；城镇常住居民人均可支配收入30458元，增长7.9%；农村常住居民人均可支配收入13631元，增长8.1%；居民消费价格上涨1.1%。

（一）坚持投资、消费、出口协同驱动，稳增长取得新成效

加大招商引资和项目建设力度。开工千万元以上项目784个，其中亿元以上项目433个，忠旺铝业86万吨高精铝一期工程、沈鼓开发区生产基地等一批重大项目建成投产。

积极培育消费热点，社会消费品零售总额实现471亿元，增长7.9%。万达广场、阿里巴巴全球货源平台等重点服务业项目投入运营，以互联网为载体，加快线上线下融合互动，全市网络零售额突破50亿元。

强化出口支撑作用，设立1000万元外贸发展专项资金，重点支持出口结构调整。全年外贸出口实现46.1亿美元，增长2%；新增自营进出口企业220余家。

（二）积极转变发展方式，结构调整迈出新步伐

加快推进现代农业发展，农业增加值实现111.8亿元，增长2.2%。新建设施蔬菜、水果基地1万亩；新增“三品”认定7.8万亩；“营口海蜇”被认定中国驰名商标，“营口对虾”、“营口柞蚕”注册国家地理标志证明商标。全市新增、规范农民专业合作社284家。粮食生产综合机械化水平达到79.1%，提高1.8个百分点。农村土地确权登记颁证30万亩，新增土地流转2.3万亩。东部振兴成果不断显现，完成移民400户，10286人稳定脱贫。

狠抓以企业为核心的工业运行，推进产业迈向中高端，规模以上工业增加值实现728.1亿元，增长2%。编制完成《中国制造2025营口行动纲要》，大力开展“千企转型升级行动”，实施40个智能制造项目，树立10户“两化”融合标杆企业，大石桥市被确定为国家新型工业化产业示范基地。推动东盛实业等20户企业集团化发展，金辰机械等10户企业建立现代企业制度，4户企业实现“新三板”上市，实现工业企业直接融资“零”的突破。我市被确定为全省唯一的非公经济综合配套改革示范区试点市。全年实施500万元以上技术改造项目150个。

全面实施加快发展服务业四年行动计划，服务业增加值实现675.6亿元，增长6.6%。电子商务迅猛发展，辽宁北方商品交易中心、东北亚镁质材料交易中心等大宗商品现货电子交易平台交易额突破2600亿元。现代物流业蓬勃发展，我市被确定为全国综合运输服务示范城市。海滨、温泉等特色旅游持续升温，赤山风景区、北海海洋公园服务功能进一步完善，盖州市获批全国休闲农业与乡村旅游示范县。金融支撑发展能力不断增强，港口金融创新试验区建设稳步推进，辽宁北方金融资产交易中心和营口港财务公司正式投入运营；丹东银行、民安财险在营设立分支机构。全年新增存款590亿元、贷款97.7亿元，实现融资总量580亿元。

加快实施创新驱动战略，大力推进产业技术创新体系建设，开展产学研合作，促进科技成果转化，组建省级产业技术创新战略联盟和产业专业技术创新平台各2个，国家级高新技术企业达到72家。营口创业大厦、众创空间投入运营，首届电子商务创新创业大赛、“互联网+”中小企业创业创新博览会成功举办。

强力推进节能减排，严控“两高”及产能过剩行业新上项目，推广应用节能技术，实行差别化阶梯水价、气价政策，强化淘汰落后产能，大力推进减排工程建设，污染物排放总量得到一定控制。

（三）着力提升经济发展活力，改革开放开创新局面

扎实推进行政审批制度改革，取消和下放行政审批权限625项，市行政审批服务中心成为我省首家列入国家社会管理和公共服务标准化试点单位。积极推进事业单位改革，出台了《分类推进事业单位改革实施意见》。深化国资国企改革，组建企业集团11家，在全省率先实现经营性国有资产集中统一监管和政企脱钩。积极推进“一照一码”等商事制度改革，新发展各类市场主体2.6万户。实施开发区（园区）与行政区管理体制改革，开发区（园区）由37个减少到26个，清理、规范中介组织22家，鲅鱼圈区获批国家中小城市综合改革试点。大力推进投融资体制改革，设立股权（创业）投资引导基金，实施政府与社会资本合作项目2个，投资总额35.1亿元。

积极融入“一带一路”战略，现已开通营口港至莫斯科等中欧直达国际班列6条，全年完成集装箱运量2.5万标准箱；辽鲁陆海货运大通道稳定运行，“营满欧”大陆桥竞争力日益凸显。中韩自贸示范区建设开始启动，韩国商品交易中心一期投入运营，综合保税区已报国务院待批。设立口岸管理局，推进大通关建设，口岸通关协作便利化水平明显提高。全年港口货物吞吐量实现3.38亿吨，增长2.3%；集装箱运量592.2万标准箱，增长5.5%。

全面加强园区建设，六个重点园区新开发土地面积10.8平方公里，固定资产投资占全市的55.2%；一般公共预算收入占全市的41.2%；千万元以上开工项目占全市的43.5%。

积极支持实体经济发展，减免小微企业税款1.05亿元；扩大“营改增”试点范围，直接减税5.6亿元；取消、减免和缓征行政事业性收费2.5亿元。

（四）完善提升城市功能，城乡面貌发生新变化

加快城市基础设施建设，营盖公路改造完成，新建改造智胜街、市府路、清华路等主城区道路5条，对重点路口、路段实施拓宽改造和单向通行，交通拥堵压力进一步缓解。实施了辽河公园景观恢复提升工程。城市绿化亮化水平进一步提升。美丽乡村建设任务全面落实，4个宜居示范镇和19个宜居示范村建设取得成效，对178个行政村的环境进行了集中综合整治，为370个村修建硬化路面357公里、砂石路面1600公里，省政府村内道路建设三年任务一年完成。

全面开展文明城市创建活动，加大市容环境综合整治力度，马路市场、非法广告、“三乱”等城市顽疾得到进一步治理。加快推进“智慧营口”建设，主城区地下管线普查和管理系统搭建工作基本完成，数字营口地理空间框架、基础地理信息数据库和应用示范系统初步建成，城市基础数据库及公共信息平台上线运行，城市管理智能化水平不断提升。

深入实施“青山、碧水、蓝天”工程。治理生产矿山55个、1016.5亩。扎实做好流域水污染防治，污水收集管网建设进一步完善，污水处理能力不断提升，城市生活垃圾处理厂基本建成。加强农业源减排，完成93家规模化畜禽养殖场粪污治理工程，创建省级生态乡镇2个、生态村20个。拆除燃煤小锅炉81台，注销老旧车及黄标车6664辆。

（五）建立健全公共服务体系，民生福祉有新提升

在财政收支矛盾突出的情况下，最大限度压缩政府一般性支出，竭尽所能增加民生投入，全年达到130.8亿元。城区道路畅通、养老服务体系提升等8项重点民生工程、35个子项全部完成年度计划任务。

全年城镇实名制就业4.5万人，城镇登记失业率2.67%，农村剩余劳动力转移就业1.9万人，零就业家庭继续保持动态为零。

全市养老、医疗、失业、工伤、生育保险参保人数持续增长，企业退休人员基本养老金标准提高到月人均1869元，城镇居民医疗保险财政补助标准达到每人每年380元，城乡居民养老保险基础养老金提高到每人每月85元，社会保障能力进一步增强。

各级各类教育协调发展，教育资源整合全面启动，营口高中迁入新址，辽师大附中营口分校建成开学，大石桥市、鲅鱼圈区、站前区顺利通过国家义务教育均衡发展评估认定。在省内率先开展新农合及大病保险异地就医报销结算和异地核查，异地新建的市第一专科医院投入运行，17所民营乡镇卫生院回归公益性。公共文化服务标准化、均等化水平不断提升，营口西大街被命名为省级历史文化街区，群众文化体育活动广泛开展。市食品安全检验检测中心实验室投入使用，乡镇农产品检验室实现全覆盖，食品药品安全形势稳中向好。

（六）加强民主法治建设，和谐营口有新进步

认真贯彻落实市人大及其常委会的各项决议决定，自觉接受人大依法监督和政协民主监督，积极听取各民主党派、工商联、无党派人士和各人民团体的意见。171件人大代表建议、287件政协提案全部办复。

深入实施“六五”普法，推进立体化社会治安防控体系建设，依法严厉打击各类违法犯罪，社会治安更加有序。着力抓好安全生产，安全生产形势持续平稳。扎实开展领导干部接访包案、信访积案化解等专项活动，社会保持和谐稳定。全面加强应急管理、气象和防震减灾工作，预防和应对突发公共事件的能力显著增强。

扎实开展“三严三实”专题教育和“解放思想大讨论”活动，政风行风进一步好转。不断规范政府重大行政决策程序，加大政务公开、政务督查和效能监察力度，政府决策力和执行力明显提高。严格落实党风廉政建设责任制，积极打造风清气正、干事创业的良好政治生态。

双拥共建、国防教育、海防海权、民兵预备役和人民防空工作深入开展；人口和计划生育、统计、新闻出版、国家安全、民族宗教、外事侨务、史志、档案等工作不断加强；慈善、红十字、老龄、妇女儿童、残疾人等各项事业取得新成绩。

各位代表：“十二五”时期是营口经济发展较快、发展质量较高、人民群众得到实惠较多的历史阶段，营口在振兴发展的道路上迈出了坚实步伐。但是，“十二五”规划部分指标没有实现，其主要原因：一是规划编制之初，正处于经济周期的波峰阶段，部分指标预期偏高。二是规划执行中，我国经济进入新常态，增速换挡是经济规律使然。三是“十二五”期间国家宏观政策调整，发展方向和支持重点发生变化。四是主动转变发展理念，实事求是做实经济数据，更加注重经济发展的质量和效益，进一步夯实持续健康发展的基础。

虽然没有全面实现预期目标，但我市经济运行总体态势是好的，经济总量在全省第4的位次没有变，发展长期向好的基本趋势没有变，持续增长的良好支撑基础和条件没有变，结构调整优化的前进态势没有变，群众生活条件不断改善的进程没有变。

各位代表：过去五年营口的发展变化来之不易。这是省委、省政府和市委正确领导的结果，是市人大、市政协监督支持的结果，是全市广大干部群众共同努力的结果。在此，我代表市政府，向辛勤工作在各条战线上的全市广大干部群众致以崇高的敬意！向给予政府工作大力支持的人大代表、政协委员，向各民主党派、工商联、无党派人士和各人民团体，向中省直单位、驻营部队、武警官兵，向所有关心、支持营口发展的各界人士表示衷心的感谢！

在肯定成绩的同时，我们也清醒地看到，全市经济和社会发展中依然存在一些亟待解决的困难和问题：经济稳中向好的基础还不牢固，影响科学发展的体制机制瓶颈依然没有有效破解；新兴产业比重低，部分行业产能过剩，经济结构仍需优化；财政支撑能力弱，收支矛盾突出，财政领域仍存在风险隐患；生态承载力下降，环境保护、节能减排压力巨大；民生改善任务繁重，社会事业发展存在短板，回迁安置、信访稳定等方面的问题没有得到根本解决；科技创新人才相对匮乏，市场活力不足，营商创业环境有待进一步优化；部分公职人员不愿作为、不敢作为、不会作为，缺乏敢闯敢试、敢于担当、勇于创新的精神。这些问题必须勇于面对，深刻反思，对症施策，切实解决。

二、“十三五”时期发展目标和主要任务

各位代表：“十三五”时期是全面建成小康社会的决胜阶段，是推动营口新一轮振兴发展的关键时期。全市经济社会发展的总体要求是：认真贯彻落实党的十八大和十八届三中、四中、五中全会精神，按照“五位一体”总体布局和“四个全面”战略布局，贯彻“四个着力”要求，统筹“四个驱动”共同发力，坚持稳中求进总基调，以建设沿海经济强市为目标，以提高发展质量和效益为中心，以产业升级、城市转型和民生改善为重点任务，加快振兴发展步伐，把营口建设成为具有较强竞争力的新型产业基地、东北亚现代化港口与物流重要城市，确保如期实现全面建成小康社会目标。

破解发展难题，厚植发展优势，全面建成小康社会，必须创新发展理念，明确发展目标和发展重点。

坚持发展第一要务，推动振兴发展。坚持以经济建设为中心不动摇，全心全意谋发展，在做优经济发展质量和效益的前提下，以存量稳增长，以增量调结构，在适度扩大总需求的同时，加快推进供给侧结构性改革，优化产业结构，做强经济实力。“十三五”时期，地区生产总值年均增长7%；规模以上工业增加值年均增长7%；固定资产投资年均增长6%；一般公共预算收入年均增长6%。力争经济发展的速度、质量和效益走在全省前列，成为东北老工业基地振兴的排头兵。

坚持加快动力转换，推动创新发展。加快实施创新驱动战略，着力推动经济发展方式从要素驱动向创新驱动转变，积极营造大众创业、万众创新氛围，让创新在全社会蔚然成风。到“十三五”期末，新技术、新产业、新业态快速发展，全民受教育程度和创新人才培养水平明显提高，以企业为主体的技术创新体系初步形成，自主创新能力全面提升，基本形成创新型经济体系和创新发展模式。

坚持问题导向，推动协调发展。更加注重发展的整体性和协调性，以沿海带动全局、西部带动东部、城市带动农村，促进新型工业化、信息化、城镇化、农业现代化同步发展，着力破解城乡区域、经济社会、经济结构等不平衡问题。到“十三五”期末，城乡一体化步伐加快，常住人口城镇化率达到70%以上。产业结构不断优化，民营经济占地区生产总值比重进一步提升。社会各项事业全面进步，区域协调发展机制基本形成。

坚持生态优先，推动绿色发展。坚持节约资源、保护环境，坚定不移地走生产发展、生活富裕、生态良好的文明发展道路，加快建设资源节约型、环境友好型社会。到“十三五”期末，大气环境质量进一步改善，能源利用效率不断提高，单位地区生产总值能耗下降和主要污染物排放量削减达到省控制要求，森林覆盖率达到45.2%，污水、垃圾处理能力全面提升。

坚持抢抓战略机遇，推动开放发展。紧紧抓住国家新一轮东北振兴和“一带一路”等战略机遇，以港口为核心战略资源，加快构建开放型经济体系。到“十三五”期末，政府职能加快转变，市场机制更趋完善，各类市场主体共同发展，外贸出口、引资引智、引进技术管理有新突破，发展潜力得到充分释放，成为东北地区最具发展活力的城市。

坚持统筹当前长远，推动共享发展。要立足当前，保障民生投入，确保经济下行压力不向民生传导。要谋划长远，保基本、兜底线、补短板，用发展成效体现共享发展理念，不断提升全市人民的生活质量和幸福指数。到“十三五”期末，保障水平不断提高，城乡基本社会保障实现全覆盖。社会就业更加充分，人民群众安居乐业。到2020年，居民收入比2010年翻一番，3.86万贫困人口全部稳定脱贫，30个贫困村全部“摘帽”。

三、2016年主要工作任务

各位代表：今年是“十三五”规划的开局之年，挑战与机遇并存。纵观国内外经济形势和发展趋势，世界经济仍处在深度调整期，经济复苏乏力；我国发展仍然面对诸多矛盾叠加、风险隐患增多的严峻挑战，经济发展环境不容乐观。但要看到，我们的发展面临着一系列重大历史机遇和有利条件，融入“一带一路”发展战略、深入实施新一轮东北振兴战略等重大举措，为我们持续平稳健康发展开拓了更大的空间。尤其是营口良好的政治生态、浓厚的发展氛围、宽松的创业环境以及全市上下昂扬向上的精神状态，必将成为我们勇往直前的强大动力。我们要准确把握战略机遇期内涵的深刻变化，适应新常态、把握新常态、引领新常态，更加有效地应对各种风险和挑战，实现“十三五”时期营口经济社会发展的良好开局。

全市经济社会发展的主要预期目标是：地区生产总值增长6%左右；固定资产投资增长5%；一般公共预算收入增长3%；社会消费品零售总额增长9%；城镇、农村常住居民人均可支配收入分别增长7%、7.5%；居民消费价格涨幅控制在2.5%以内。

围绕实现上述目标，我们要重点做好以下工作：

（一）加大招商引资和盘活存量力度，着力推进经济持续稳定增长

更加突出招商引资，坚持“走出去”与“请进来”并重，以现有存量企业为依托，实施以商招商、产业链招商。积极引进新能源、新材料、先进装备制造业及现代服务业项目，补齐产业短板。深化与跨国集团和行业领军企业的战略合作，着力引进一批调结构、促转型、增后劲的重大项目。加大对公共医疗、教育培训、公共文化设施等与群众生活息息相关领域的招商力度，增强公共产品和服务的有效供给能力。全年开工千万元以上项目力争超过500个，其中亿元以上项目突破260个。

把盘活存量作为促进发展的重要措施，积极整合和优化各类存量资源，为经济稳定增长提供新空间。通过兼并重组、产权转让等方式，对产能过剩行业、空壳企业进行清理，提高资源配置效率。积极开展环保违规建设项目清理整顿，通过备案完善一批、整顿规范一批、依法关闭一批，对已建成或在建、停建的企业依法进行规范，减少社会投资浪费，盘活投资存量，实现环境保护和经济社会共赢发展。

大力推进去库存，严控房地产土地供给，通过政策引导，吸引外来人口落户入住，有序推进农民市民化，全面实行棚户区改造货币化安置，培育住房租赁市场，消化房地产库存，稳定房地产市场。继续优化生产要素供给，加大闲置土地清理整顿力度，提高投资强度，推进低效用地用海再开发利用，千方百计提高水、电、气等要素利用效率和供应能力。积极优化公共资源配置，发挥市场在资源配置中的决定性作用，强化公共事业、社会事业、国有企业等公共资源的管理和利用，着力提高配置效率和公平性。

（二）深化各项改革，着力完善体制机制

持续推进简政放权。积极承接国家和省下放的行政职权事项，再精简、清理、规范一批审批事项，为大众创业、万众创新减负清障。拓展网上并联审批系统，优化企业注册、重大投资项目等跨部门审批业务流程，提高审批效率。落实“权力清单、责任清单”制度，确保行政权力规范运行。

深化国资国企改革。完善国有企业薪酬分配、产权管理、财务监督等配套制度，提升管理水平，巩固改革成果。实施分类监管，完善法人治理结构，确保国有资产保值增值。

推动农村综合改革。全面完成90万亩农用地确权登记颁证任务，新增土地流转面积3万亩以上。全面落实良种补贴等惠农政策，农业保险投保面积96.2万亩。深化集体林权制度改革，鼓励集体林地经营权、使用权和林木所有权流转，林业保险面积达到240万亩。

深化财税体制改革。落实税收保障办法，大力培育税源，增加地方可用财力。完善预算绩效管理体制，建立评价结果与预算安排、资金拨付相结合的激励约束机制。建立健全债务管理制度和风险预警处置机制，严控政府性债务风险。

深化投融资体制改革。推广政府与社会资本合作等投融资模式，加快建设城市停车场、地下综合管网等城市基础设施以及交通、水利工程。不断强化与国家开发性金融机构合作，在扶贫、基础设施和民生等领域争取更多支持。推动股权（创业）投资引导基金发展，促进财政性资金由直接投资向股权投资等市场化方式转变。

（三）加快产业转型升级，着力推进结构调整

大力发展现代农业和县域经济。加快农业结构调整，着力培育新型农业经营主体，促进农业发展方式转变。开展农业示范园区创建活动，创建高标准示范园区20家，新增“三品”认定面积10万亩，规范农民专业合作社不低于100家。积极实施品牌提升计划，力争在品牌创建、农产品出口、综合机械化等方面实现新的突破。积极培育县域优势特色产业，提升县域工业园区投入产出效益，促进县域经济发展。

推动工业结构优化升级。着力实施《中国制造2025营口行动纲要》，加快“两化”融合和大数据平台建设，积极构建以智能制造为引领的新型工业体系。继续实施“千企转型升级行动”，引导扶持300户企业开展装备工艺技术改造和新产品开发，提升企业竞争力。设立促进产业发展基金和投资（创业）引导基金，推动现有六大主导产业转型升级改造和高新技术嫁接升级改造，促进高端装备制造、节能环保、海洋经济等新兴产业发展。推动省非公经济综合配套改革示范区试点市建设，实现非公经济新发展。继续加大对企业帮扶力度，促进产品有市场、有效益的阶段性停产企业尽快开工复产、脱困增效。实施涉企收费清单管理，落实惠企政策，降低企业生产成本。全年新增规模以上工业企业30家，规模以上工业增加值增长5%。

加快发展现代服务业。实施“互联网+流通”行动计划，突出抓好物流园区建设，争创国家级电子商务示范基地，实现网络和实体经济融合发展。全面发展普惠金融，着力解决小微企业和“三农”融资难、融资贵问题。积极推进金融创新，大力发展港口、科技和互联网金融，加快北方金融资产交易中心发展。全年新增金融机构3家，新增主板上市和“新三板”挂牌上市企业10家以上。扩大旅游产业区域合作，完善旅游基础设施、服务设施，提升旅游业发展品质。以目的地旅游项目为支撑，加快发展观光、休闲、度假等旅游业态，鲅鱼圈区积极争创省级旅游度假区。整合河海、老街等资源，打造滨河临海旅游观光带。

狠抓节能降耗与淘汰落后产能。推进各项节能减排工程建设，落实结构减排各项措施，严格控制动态增量，推进重点用能企业节能降耗。加快农业源减排项目建设，加大黄标车、10吨以下燃煤锅炉以及高污染、高耗能等落后产能淘汰力度，全面完成国家和省下达的节能减排任务。

（四）培育发展新引擎，着力鼓励创新创业

强化企业创新主体地位。实施科技企业培育工程，增强企业在创新驱动发展中的主体作用。推进产业技术创新战略联盟建设，鼓励企业与高校、科研院所合作进行技术攻关。加快技术创新项目建设，培育市级以上企业技术中心14个、工程技术中心20个。

加强科技创新引导。强化科技支撑引领，搭建科技创新网络综合服务平台，继续推进汽保研发检测、装备制造研发等共性技术平台建设。完善科技成果转化机制，对企业购买技术或联合研发实现的专利转化项目予以支持。

积极引进科技人才。健全科技人才培养选拔、评价使用、流动配置、创新激励等机制，激发人才创新创造活力。依托重大项目、重点企业、科技园区和特色优势产业，大力培养和引进高层次科技人才、高水平创新团队。

着力营造创业氛围。完善创业服务体系，进一步降低创业门槛和创业成本。出台支持创新创业的资金扶持政策，鼓励社会力量创办创业孵化基地，支持众创空间发展，加快形成创新带动创业、创业促进创新的体制机制。

（五）聚焦民生福祉，着力保障和改善民生

积极稳定就业。进一步拓展就业空间，全年实名制就业2.6万人，城镇登记失业率控制在3.5%以内。抓好高校毕业生、城镇就业困难群体和农村转移劳动力就业，高校毕业生就业率保持90%以上，困难家庭高校毕业生就业率达到100%。

不断完善社会保障体系。继续提高企业退休人员养老金、城乡居民基础养老金标准和城镇居民基本医疗保险政府补助标准，推行医疗救助“一站式”即时结算服务。全市城乡最低保障标准平均提高7%以上，基本完成社会保障“一卡通”工程。加快发展养老产业，积极推进智慧养老、居家养老，完成市养老服务中心一期建设工程，促进养老服务机构规范发展。

加大民生工程实施力度。把全市人民过上美好幸福生活的新期待作为工作出发点和落脚点，实施好保障性住房、校园食品安全、城市新能源公交等10件民生实事。突出精准扶贫、精准脱贫，对贫困群众实施分类帮扶，全年完成脱贫1万人、移民150户，努力提高全市人民的幸福感和满意度。

大力发展社会各项事业。促进学前教育普及普惠发展，学前三年入园率达到96%；推进义务教育均衡发展，六个市（县）区全部通过省级认定；加快基础教育改革和发展，实施教育教学质量提升工程，建立校长、教师定期培训制度，全力整合教育教学资源。改善市特教学校办学条件，异地新建高职院校区，整合、改建中职学校，进一步理顺营口理工学院体制机制。深化医药卫生体制改革，突出抓好医疗服务、公共卫生和基本药物制度落实，全科医师培养基地全面建成；继续完善新农合管理制度和支付方式改革，新农合异地报销扩大至全国范围。大力发展文化产业，启动省级公共文化服务体系示范项目建设，完善图书大厦功能，力争年底前投入使用。创新群众体育工作机制，完善全民健身场地建设，广泛开展全民健身活动。推进食品安全放心市创建工作，确保舌尖上的安全。

深入开展平安营口建设。启动实施“七五”普法，推进法治营口建设。创新完善社会治安防控体系，增强防范控制能力，提升群众安全感。健全完善社会稳定风险评估机制，依法规范信访工作秩序，及时解决群众合理诉求。坚守安全红线，严格落实安全生产责任，强化隐患排查整治，保持安全生产形势持续稳定。

（六）融入“一带一路”战略，着力推进新一轮高水平对外开放

抢抓开放发展新机遇。深度对接“一带一路”、中蒙俄经济走廊等重大发展战略，积极参与京津冀协同发展。充分发挥港口的重要作用，拓展外贸航线，努力提升港口通行能力，不断增加港口货物吞吐量。推进国际贸易“单一窗口”建设，改善通关环境。加快推进综合保税区、营口中韩自贸示范区建设，拓展营口港与俄罗斯铁路总公司物流中心业务，完善“营满欧”大陆桥基础设施与配套设施，打造“中欧班列”品牌，把营口建成辐射东北、内蒙古、中亚、中东欧等国家和地区的贸易和商品集散地。鼓励优势企业“走出去”，扩大境外投资，开展境外资源开发、国际工程建设。

着力提升园区对经济增长的贡献率。继续加大重点园区开发建设力度，完善配套服务功能，加快促进产能释放，提升园区发展质量。创新园区体制机制，完善园区招商服务功能，增强园区发展活力。

努力扩大外贸出口。充分发挥外贸发展专项资金作用，重点支持出口品牌建设，扩大出口信用保险覆盖面。推广“互联网+外贸”服务模式，支持阿里巴巴全球货源平台、迪威视讯等交易平台开展跨境业务。全年外贸出口增长8.5%。

（七）统筹城乡发展，着力打造生态宜居城市

强化环境综合整治。实施蓝天工程，下大气力做好大气污染防治，强化综合治理措施和重污染天气应急管理，重点抓好清洁生产、扬尘污染管控，实现PM2.5、PM10双下降。实施碧水工程，重点启动大旱河、沙河、熊岳河等劣五类入海河流整治，加强流域内涉水污染源监管，确保污染治理设施稳定达标运行。开展县级以上集中式饮用水水源地环境综合整治，消除水源地环境污染，确保饮用水安全。实施青山工程，加快推进工矿废弃地复垦、矿区生态和地质环境整治。实施沃土工程，加大耕地保护力度，加强农村土地综合整治，建立健全耕地质量标准体系和长效管理机制，不断提升农业可持续发展能力。实施城市环境治理工程，加快污水、污泥、存量垃圾处理整治。

完善城市基础设施。加大老城区公共设施和基础配套设施建设力度，推进停车场、菜市场、公共绿地、市民健身广场等工程建设。新建东海大街、滨河街西段等4条道路，对盼盼路、学府路进行维修改造。加快推进仙人岛港区疏港铁路、鲅鱼圈港区和仙人岛港区疏港公路以及码头、泊位等港口基础设施建设，兰旗机场实现通航。

积极稳妥推进新型城镇化。进一步完善发展规划，优化城市布局，加快新城、新市镇基础设施建设，着力提升发展质量和内涵。继续开展农村环境集中综合整治，完善农村环境治理和管护体系，建设美丽乡村。全面放开城区和建制镇落户限制，实施差别化落户政策，引导农业人口有序转移。

深入实施东部振兴战略。加大政策、资金、人才等要素向东部山区倾斜力度。积极扶持水果、蔬菜等主导产业发展，加快开发乡村旅游、沟域经济，打造一批特色产业乡（镇）村。加强农田水利、道路交通等基础设施建设，强化地质灾害预警，不断改善农村生产生活条件。加强农村科教文化事业发展，提高公共服务水平。千方百计增加农民收入，全年东部山区农民收入人均增加不低于1000元。

加强国防教育、民兵预备役建设，深化军民融合发展，支持军队改革，积极开展双拥共建活动，深化文明城市创建。做好统计、物价监管、人民防空、人口和计划生育、新闻出版、国家安全、民族宗教、外事侨务、史志、档案、气象、地震、海防海权等工作。积极发展慈善、红十字、老龄、妇女儿童、残疾人、未成年人等各项事业。

各位代表，加强政府自身建设，是完成全市经济社会发展各项任务的重要保证。我们将按照“三严三实”的要求，不断规范政府行政行为，提高行政效能。

建设法治政府。坚持法定职责必须为、法无授权不可为，依法全面履行政府职能。加强政府立法制度建设，建立健全规章制定程序。建立重大决策审查机制，严格执行公众参与、专家论证、风险评估、合法性审查和集体讨论决定等法定程序。自觉接受人大依法监督、政协民主监督，认真听取各民主党派、人民团体和各界人士的意见建议，加大政务公开力度，主动接受社会监督、舆论监督。

建设服务政府。牢固树立执政为民的理念，增强主动服务意识，创新服务管理方式，不断提高工作效率和服务水平。持续抓好作风建设，坚决纠正不作为、乱作为，坚决克服懒政、怠政，坚决惩处失职、渎职。

建设诚信政府。严格履行政府各项承诺，做到言必信、行必果，切实增强政府公信力。加快建立以政务诚信、商务诚信、社会诚信为主体的社会信用体系，完善信用奖惩制度，积极营造良好的信用环境。

建设责任政府。始终坚持对党和人民事业高度负责的态度，深入落实政府部门责任制和岗位责任制，建立科学合理的政府绩效评估指标体系和评估机制。崇尚实干，狠抓落实，强化督查，确保每项工作都落到实处、取得实效。

建设廉洁政府。认真履行党风廉政建设责任制，加强重点领域、关键环节的行政监察和审计监督，强化公共资源交易监管。从严管理政府工作人员，用制度管住权力、预防腐败。坚决查处违法违纪案件，以“零容忍”的态度惩治腐败，积极营造良好的政治生态和从政环境。

各位代表：营口已经站在新的历史起点上，我们要肩负起振兴发展的使命，担当起为民谋利的职责。让我们在省委、省政府和市委的正确领导下，紧紧依靠全市人民，凝心聚力、攻坚克难、真抓实干，为加快营口振兴发展，全面建成小康社会而努力奋斗！
